# CYP3A Activity in End-of-Life Cancer Patients Measured by 4β-Hydroxycholesterol/cholesterol Ratio, in Men and Women

**DOI:** 10.3390/cancers13184689

**Published:** 2021-09-18

**Authors:** Helena Bergström, Maria Helde Frankling, Caritha Klasson, Anita Lövgren Sandblom, Ulf Diczfalusy, Linda Björkhem-Bergman

**Affiliations:** 1 Department of Neurobiology, Care Sciences and Society (NVS), Division of Clinical Geriatrics, Karolinska Institutet, Blickagången 16, Neo Floor 7, SE-141 83 Huddinge, Sweden; maria.helde.frankling@ki.se (M.H.F.); caritha.klasson@ki.se (C.K.); linda.bjorkhem-bergman@ki.se (L.B.-B.); 2 Department of Cancer, Section of Head, Neck, Lung and Skin Tumors, Karolinska University Hospital, Eugeniavägen 11, SE-171 76 Stockholm, Sweden; 3 Department of Palliative Medicine, Stockholms Sjukhem, Mariebergsgatan 22, SE-112 19 Stockholm, Sweden; 4 Department of Laboratory Medicine, Division of Clinical Chemistry, Karolinska Institute, SE-141 52 Stockholm, Sweden; anita.lovgren.sandblom@ki.se (A.L.S.); ulf.diczfalusy@ki.se (U.D.); 5 Department of Clinical Chemistry, Karolinska University Laboratory, Karolinska University Hospital, SE-141 86 Huddinge, Sweden

**Keywords:** palliative care, cancer, CYP3A-activity, end-of life, 4β-hydroxycholesterol/cholesterol, 25-hydroxyvitamin D

## Abstract

**Simple Summary:**

The elimination of drugs by enzymes in the liver may be impaired in cancer patients that are close to death (end-of-life). This could cause unwanted side effects or lack of effect of drugs and compromise the quality of life in patients. Blood samples collected in 137 deceased end-of-life cancer patients were analyzed for the marker 4β-hydroxycholesterol/cholesterol (4β-OHC/C), representing the activity of the most important drug eliminating enzyme, CYP3A. In addition, samples from young (*n* = 280) and elderly (*n* = 30) controls were analyzed for 4β-OHC/C. The average 4β-OHC/C was higher in male and female end-of-life cancer patients than in young and elderly controls without cancer. This finding may suggest that the ability to eliminate drugs by CYP3A is maintained until end of life and that drugs metabolized by CYP3 may not need dose adjustment or discontinuation in cancer patients close to death.

**Abstract:**

More than 50% of all drugs are metabolized by the cytochrome P450 3A enzyme (CYP3A). The aim of this study was to investigate if the CYP3A activity, measured by the endogenous marker 4β-hydroxycholesterol/cholesterol ratio (4β-OHC/C), is changed during the last weeks and days of life in men and women. To this end, serum samples from 137 deceased patients (median age 70 years) collected at a single time point 1–60 days before death, were analyzed and compared to 280 young (median 27 years), and 30 elderly (median age 70 years) non-cancer controls. There were no significant differences in the 4β-OHC/C ratio between men and women in end-of-life patients (*p* < 0.25). The median 4β-OHC/C was significantly higher in end-of-life male patients compared to both young (*p* < 0.0001) and elderly (*p* < 0.05) male controls. In a similar manner, 4β-OHC/C in end-of-life female patients was significantly higher compared to young and elderly female controls, *p* < 0.0001 and *p* < 0.001, respectively. There was no significant correlation between 4β-OHC/C and survival time. The results from this study suggest maintained CYP3A activity to the very last days of life and even a capacity of induction of the enzyme in end-of-life cancer patients.

## 1. Introduction

The capacity to metabolize drugs during the last weeks and days of life in cancer patients is sparsely studied. Knowledge about drug metabolism in these patients may be of importance for when treatment should be discontinued or doses adjusted. Predicting individual metabolic profiles by using a phenotypic marker could allow for making individual dose adjustments or choosing other drugs, thus optimizing treatment. In addition, an impaired drug metabolism could cause unwanted side effects or lack of effect and impair quality of life (QoL).

More than 50% of all clinically used drugs are metabolized by the cytochrome P450 3A enzyme (CYP3A) [1]. In addition, CYP3A is also important for the metabolism of various endogenous substance synthesized in the human body, including steroid hormones, vitamin D, etc. CYP3A is present in both liver and intestine [1]. To measure CYP3A-activity in humans, probe drugs may be used, and midazolam clearance is considered the “golden standard” [2]. However, in vulnerable populations, such as elderly or end-of-life patients, exogenous probe drugs could cause more harm than benefit. Instead, endogenous markers of CYP3A activity can be used, e.g., 4β-hydroxycholesterol/cholesterol ratio (4β-OHC/C) in blood. Studies have shown that 4β-OHC/C is as good as midazolam clearance in detecting rapid induction in CYP3A, while comparisons in the basal state have shown diverging results [3,4]. A recent study indicates that 4β-OHC/C does not reflect intestinal CYP3A activity, which may explain this inconsistency [5]. However, 4β-OHC/C does have several advantages, like low-intra individual variability and a long half-life of 17 days, resulting in stable plasma concentrations [6]. Due to its long half-life, 4β-OHC/C is less suitable for detecting rapid changes, especially during inhibition of CYP3A by other drugs [7].

Phenotyping studies on CYP3A metabolism in palliative cancer patients have usually been performed using exogenous probes. Studies evaluating the CYP3A activity using midazolam or erythromycin breath test in cancer patients have shown reduced enzymatic activity compared to healthy volunteers [8,9]. However, the subjects in these studies with advanced, non-curable cancer had normal BMI and did not suffer from cachexia or organ failure; common denominators in end-of-life patients [10,11]. Furthermore, there are few studies of CYP3A activity in cancer patients using endogenous probes, and to our knowledge none in end-of-life cancer patients [12,13]. Thus, there is still a lack of information concerning CYP3A metabolism at the very end of life, the last weeks and days. 

Previous studies have shown that 25-hydroxyvitamin D (25-OHD) may induce CYP3A-activity. However, results have been discordant [14,15]. Many cancer patients suffer from vitamin D deficiency and vitamin D levels appear to decrease closer to death [16]. If vitamin D deficiency does affect CYP3A-activity, late-stage cancer patients may be at risk of reduced capacity to metabolize drugs.

The primary aim of this study was to investigate if CYP3A activity, measured by the endogenous marker 4β-OHC/C, is changed in end-of-life cancer patients compared to young and elderly non-cancer controls, with a focus on sex differences. Secondly, we wanted to study a possible correlation between 4β-OHC/C and survival time, albumin, C-reactive protein (CRP), and 25-OHD.

## 2. Materials and Methods

In this retrospective, observational study, we used serum samples from deceased patients included in two previous studies performed in three palliative care units in Stockholm, Sweden. Only patients with a single point serum sample collected ≤60 days before death were included. All patients had given their written informed consent to participate in the original studies and that their samples would be stored in a biobank for future analysis.

The three advanced medical home care units ASIH Stockholm Södra, ASIH Stockholm Norr, and ASIH Stockholms Sjukhem cover different parts of the Stockholm region and offer advanced palliative and supportive medical home care in the patient’s own home. In addition, all three units have in-patient Hospice Wards of 16, 12, and 42 beds, respectively. The medical care in these units is financed by the state.

The first study was a randomized, placebo-controlled, double-blind study on vitamin D in palliative care, “The Palliative-D study” performed during 2017–2020 [17,18,19]. Serum samples from the screening visit were used, i.e., before any study drug had been given. Patients with advanced cancer, enrolled at any of the three different palliative home care units, without hypercalcemia during the last 2 months and with eGFR > 30 mL/min, were eligible. 

The second study was a cross-sectional, observational, single-center study, performed at the in-patient Hospice Ward at ASIH Stockholm Södra with the aim to study the immune system in end-of-life patients and its possible relationship to vitamin D, measured as 25-OHD. The inclusion criteria for the study were written informed consent, advanced cancer, and being admitted to the Hospice Ward. Samples from 37 patients collected ≤60 days before death were available for the present study. 

In addition, 30 elderly controls were submitted to sampling in a similar manner. Inclusion criteria for the elderly controls were no ongoing infection, no ongoing cancer disease, and no ongoing treatment with drugs known to be strong inducers or inhibitors CYP3A activity. Finally, previously collected data from young volunteers (*n* = 280) were used for comparisons with those from the patients and elderly controls. Further details can be found in the original studies [20,21].

CRP, albumin, creatinine, and 25-OHD were analyzed by the Laboratory of Clinical Chemistry, Karolinska University Hospital, Sweden with ISO 15189:2012 accredited methods. For subjects in the immune study and for all controls, cholesterol was determined by a commercial enzymatic method (CHOD PAP, Roche Diagnostics GmbH, Mannheim, Germany) using a Roche Modular P800 instrument. A different method for cholesterol analysis demanding smaller volumes of plasma was chosen for the palliative study cohort and is described in detail below. Internal controls have found the methods to be comparable. 

Cholesterol was determined by isotope-dilution gas chromatography-mass spectrometry. In short, 5 µL of serum or plasma was pipetted into a test tube with a Teflon-lined screw cap. A total of 2 µg of [2H6] cholesterol (internal standard) was added. Alkaline hydrolysis was carried out by addition of 1 mL of 1M NaOH in 90% ethanol, a short vigorous stir, and subsequent incubation at 65 °C for 1 h with continuous agitation. Water (0.5 mL) was added, and the sample was extracted twice with 3 mL of cyclohexane. 

The combined cyclohexane phases were evaporated to dryness and derivatized with 200 µL pyridine/hexamethyldisilazane/chlorotrimethylsilane (3/2/1, *v*/*v*/*v*). After incubation at 60 °C for 30 min, the samples were evaporated to dryness and dissolved in 200 µL hexane. The sample (2 µL) was injected into a Hewlett Packard HP6890 gas chromatograph connected to a Hewlett Packard HP 5973 mass selective detector. The gas chromatograph was equipped with an HP5 MS column (30 m × 0.25 mm, 0.25 µm film thickness). The temperature program started at 180 °C where it was held for 1 min, increased by 20°/min until 250 °C where the rate was changed to 3.3°/min until a final temperature of 300 °C where it was maintained for 10 min. The ions *m*/*z* 458 and *m*/*z* 464 were used for detection of cholesterol and [2H6] cholesterol, respectively.

The oxysterol 4β-hydroxycholesterol was determined by isotope-dilution gas chromatography-mass spectrometry as described in [20] with the modification of the method reported in [6]. In short, 100 ng of 2H7-4β-hydroxycholesterol (internal standard) was added to 250 µL of plasma. A total of 1 mL of 0.7 M KOH in ethanol was added and the mixture was incubated at room temperature for 30 min. Phosphoric acid was added to bring the sample to a neutral pH and the sample was centrifuged at 1000× *g* for 10 min. A 30 mg Strata X solid phase extraction column (Phenomenex) was conditioned with 1 mL of methanol and equilibrated with 1 mL of water. After application of the sample to the column, the column was washed with 1 mL of 10% methanol in water. 4β-hydroxycholesterol was then eluted with 1 mL 85% acetonitrile in water. The eluate was evaporated to dryness and the residue derivatized with 100 µL tert-butyldimethylsilylimidazole diformamide. The sample was incubated at 60 °C for 2 h. After extraction with isooctane the solvent was evaporated, and the sample dissolved in 60 µL of hexane. A total of 1 mL of the hexane solution was injected into a Hewlett Packard HP6890/5973 MSD gas chromatograph-mass spectrometer as described in [20]. The coefficient of variation was 8.2% and the method was linear up to 600 ng/mL [6].

Clinical data on age, sex, diagnosis, prescribed drugs the day of blood collection, data on laboratory analyses, and survival time were extracted from the medical records in the previously performed studies. 

Statistical analyses were performed using Graph Prism v9.1.0 (221) software (San Diego, CA, USA). D’Agostino and Pearson normality test was conducted to test for Gaussian distribution of data and arithmetic means, standard deviations (SDs), medians and interquartile ranges were calculated. Statistical significance was tested using Mann–Whitney U test for variables with non-Gaussian distribution for continuous variables, and Fischer’s exact test for categorical variables. Correlation analyses were performed using Spearman’s rank test. A *p*-value < 0.05 was considered statistically significant.

All variables in patients including age, CRP, creatinine, cholesterol, 25-OHD, survival time, and 4β-OH-C/C had non-Gaussian distribution in the study cohorts. Albumin was normally distributed in female patients but not in males, and thus non-parametric methods were used. 

## 3. Results

### 3.1. Patient Study Cohort

From the first study, Palliative-D, 108 of 530 patients fulfilled the inclusion criteria for this study (i.e., death within 60 days after samples were collected). Seven patients (four men and three women) were excluded due to lack of serum. In addition, one male patient was excluded due to treatment with an antiepileptic drug that is known to cause strong induction of CYP3A-activity. Thus, a total of 100 patients, 50 men and 50 women, were eligible from this study. From the second cohort, the immune study, 37 patients fulfilled the inclusion criteria, 19 men and 18 women, thus making the total number of 137 subjects in the patient cohort for the current study. None of the five women <50 years in the cohort were judged as fertile due to previous treatment with cytotoxic agents. 

Baseline patient characteristics including age, CRP, albumin, creatinine, 25-OHD, survival time from baseline (days), cholesterol, and 4β-OHC/C ratio are presented in Table 1. The median age for all patients was 70 years (men and women). There were no significant differences between men and women in the presented variables, with the exception for creatinine. In addition, there were no significant sex differences in the occurrence of different forms of cancer, except for breast and gynecological cancers only present in females and prostate cancer only present in males (Table 1).

The most common medication in the cohort was proton pump inhibitors (PPIs) in 71% of the patients (*n* = 97). The second most common drug treatments were long-term glucocorticoids (*n* = 82) and opioids (*n* = 82), corresponding to 60% of the patients. Furthermore, 31% (*n* = 43) were treated with antihypertensive medication, 27% (*n* = 38) with benzodiazepines and 18% (*n* = 25) with nonsteroidal anti-inflammatory drugs (NSAIDs). Seven patients (5%) were treated with cytotoxic agents, and seven (5%) with antipsychotic drugs. Three male patients were treated with fluconazole, a moderate inhibitor of the CYP3A enzyme [22]. However, in a recent study of fluconazole treatment for eight days in 17 healthy volunteers, the 4β-OHC/C ratio increased during fluconazole treatment [23]. Additionally, the ratios in the patients in our study were in line with that found in the other male subjects, and therefore any effects of fluconazole on CYP3A were judged as negligible. Finally, 16 patients (11.7%) used anti-depressive medication.

### 3.2. Study Controls: Young and Elderly

The baseline data from the young controls have been previously published and are shown in Table 2 [20,21]. In the young cohort (median age 27 years), there were significant differences in cholesterol levels between men and women. This is to be expected, as lipids may fluctuate due to hormonal variations during the menstrual cycle in women [24]. There was no significant difference in the 4β-OHC/C ratio between the sexes. 

In the elderly control cohort, the median age was significantly higher in men (*n* = 15) compared to women (*n* = 15), 73 years versus 68 years. The median age for men and women together was 70 years. In addition, men had significantly higher creatinine values suggesting a lower kidney function. There were no significant differences in concomitant medications between men and women in the cohort. A total of 20% of females and 33% of males were treated with statins, while 50% had antihypertensive treatment. Additionally, 10% of the cohort were treated with PPI. Finally, there were no differences between the sexes in the 4β-OHC/C ratio.

### 3.3. 4β-OCH/C Ratio in Patients, Young and Elderly Controls

**Figure 1:**
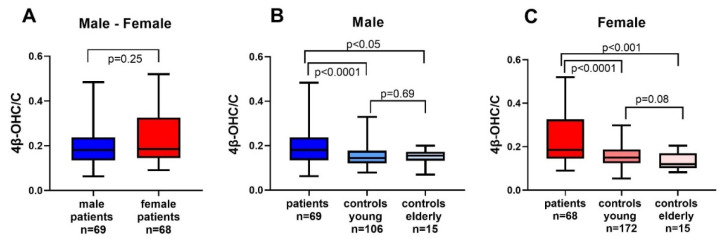
Differences in 4β-hydroxycholesterol/cholesterol ratio (4β-OHC/C) between (**A**) male and female patients (**B**) male cancer patients, young and elderly controls, and (**C**) female cancer patients, young and elderly controls.

There were no significant differences in the 4β-OHC/C ratio between male and female patients (Figure 1A). When comparing male cancer patients with healthy young or elderly male controls, there were significant differences in the 4β-OHC/C ratio (Figure 1B). The difference in the ratio between male young and elderly controls was not significant. 

There was a significant difference in the 4β-OHC/C ratio between female patients, and young or elderly female controls, respectively (Figure 1C). In addition, there was no difference in the ratio between young and elderly female controls. 

In a subgroup analysis between patients with and without glucocorticoids, there was no difference in the median 4β-OHC/C ratio (*p* = 0.72).

### 3.4. Correlation Analysis 4β-OHC/C 

**Figure 2:**
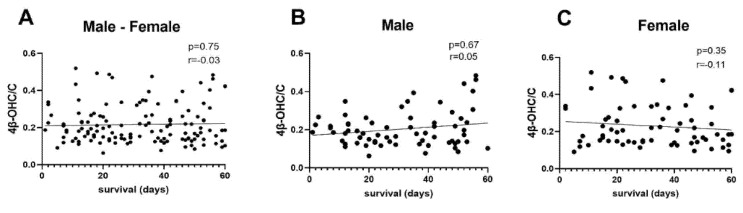
Correlation between 4β-hydroxycholesterol/cholesterol ratio (4β-OHC/C) and survival time measured in days in (**A**) all patients (*n* = 137) (**B**) males (*n* = 69), and (**C**) females (*n* = 68). Statistical analysis was performed using Spearman’s rank test. r = Spearman’s r.

There was no correlation between 4β-OHC/C and survival time in patients whether analyzed by all subjects, or male or female separately (Figure 2). 

There was no significant correlation between the 4β-OHC/C ratio and age, albumin, CRP, or 25-OHD in the patient cohort (Table 3).

## 4. Discussion

In the present study, we found significantly higher values of the endogenous CYP3A activity marker 4β-OHC/C in end-of life cancer patients, compared to healthy young and elderly controls. There were no significant differences in the ratio between males and females in the patient population, nor in the controls. In addition, we found no significant correlations between 4β-OHC/C and survival, albumin, CRP, or 25-OHD. 

Although age, the cancer disease and the different drugs used by the patients may affect CYP3A-activity, we wanted to perform an “as is” study, reflecting the overall functionality near death. Thus, the study population is representative for cancer patients in a hospice setting, i.e., a heterogenous cohort of different cancer types, ages and with ongoing medication used in palliative care. Thus, this makes the results more valid to the clinical setting.

Both age and cancer may affect lipoprotein metabolism, and thus have the potential to influence levels of cholesterol and its metabolites near death [25,26]. However, the use of the cholesterol ratio instead of 4β-OHC alone is thought to ameliorate these factors to some extent. 

The results of this study suggest a possible increase in CYP3A activity in end-of-life cancer patients with life-threatening disease, close to death and thus indicate a maintained capacity to metabolize drugs to the very last days of life. This finding is contradictory to previous studies performed in patients with advanced, non-curable cancer, suggesting a decrease in CYP3A activity in these patients [27,28]. However, these studies have mostly been performed on patients earlier in the cancer disease trajectory, with normal performance status and liver enzymes [27,29]. Translational repression of CYP3A genes, by the pro-inflammatory cytokine interleukin-6 (IL-6) and tumor necrosis factor α (TNF-α) have been suggested as the mechanisms causing decreased CYP3A-activity in cancer patients [27,30]. However, the exact mechanisms remain unclear [31]. 

In our study, 60% of the patients were on glucocorticoid treatment at time for inclusion. The increases in 4β-OHC/C ratio may be explained by an induction of CYP3A activity caused by this concomitant treatment [32,33,34]. Glucocorticoids signal via the glucocorticoid receptor (GR), acting as a transcription factor suppressing pro-inflammatory transcription factor NF- κβ [33]. In this manner an activated immune system is impaired, and inflammatory mediators like interleukins and cytokines are reduced [33]. Although not demonstrated in humans this could theoretically restore or even induce CYP3A activity by an increase in transcription of the enzyme. However, there was no difference in the 4β-OHC/C ratio between patients with or without glucocorticoid treatment in this study cohort. Interestingly, it has been suggested that GR has a key role in restoring the homeostasis in the body during critical illnesses, by regulating the stress, acute phase and tissue defense response [35]. Finally, other causes of an increase in CYP3A activity could be a dampened immune response seen in patients with advanced cancer disease [36].

In this study, there were no sex differences in cholesterol or 4β-OHC in the cancer patients or elderly controls. Higher levels of cholesterol and 4β-OHC in women were found in a previous study including elderly cancer patients (women *n* = 10, men *n* = 23), but similarly to this study, the 4βOHC/C ratio was equal in females and males [37]. Baseline variables in the cohort were comparable in men and women, except that in women 24 of 68 patients (35%) suffered from breast and gynecological cancers. Although these cancer forms probably do not affect 4βOHC/C ratio differently than other cancer forms in women we cannot fully exclude that this difference in cancer types may affect the results.

Further, the suggested association between 25-OHD and CYP3A activity [14,15] could not be confirmed in our study. This is in line with a previous study, where high-dose vitamin D supplementation to immunodeficient patients for one year did not affect the 4β-OHC/C ratio [14]. This could be explained by in vitro data suggesting that the active form of vitamin D, 1-alpha, 25-dihydroxy vitamin D3, regulates the transcription of CYP3A solely in the intestine, and not in the liver [38]. Notably, studies show that the 4β-OHC/C ratio most likely reflects only hepatic CY3A activity, and not intestinal CYP3A activity [5].

To our knowledge, this is the first study performed on CYP3A-activity measured as 4β-OHC/C in cancer patients, days to weeks before death. The study is performed as an “as is” study with no strict inclusion and exclusion criteria, thus reflecting the true clinical situation in palliative care, making the results more generalizable. In fact, there are few studies on CYP3A activity in the basal state in cancer patients, as most studies have been performed investigating cytotoxic agents where potential inducing or inhibiting effects on CYP3A has been the primary aim of the study [12,13]. Additionally, previous studies on CYP3A activity in cancer patients have been performed using exogenous probes, e.g., oral, or intravenous midazolam or erythromycin breath test [8,9]. Results have been conflicting. One study demonstrated a reduction in CYP3A activity by 40% in cancer patients compared to healthy volunteers, while another study in Asian cancer patients compared to non-cancer patients did not find any differences [8,39]. 

One limitation in this study is the lack of genotyping for single-nucleotide polymorphisms (SNIPs) in the CYP3A genes, to assess potential effects on the results. However, due to conflicting results on genotyping for CYP3A4 and CYP3A5 in cancer patients, we decided against performing these analyses [37,40,41,42]. Secondly, a significant association between 4β-OHC/C and body mass index (BMI) was found in healthy volunteers [43]. However, in our cohort of end-of-life cancer patients, measuring patients’ weight was not considered a priority during the last weeks of life and thus these data are not available. 

As the end of life approaches, many organs in the body decrease their functionality i.e., kidney and brain function [11]. The result of this study suggests that the liver can increase the CYP3A activity in the hour of need, close to death. Furthermore, this may imply that adjustment of doses of a drug in cancer patients close to death can be done without having to lower the doses, to adjust for a reduction in hepatic metabolism. Still, as the results of our study contradict previous findings of CYP3A activity in cancer, they should be interpreted with caution. 

In the future, this study should be repeated, preferentially with a larger sample size of end-of-life cancer patients. We also suggest focusing on 3 to 4 different types of cancer, to reduce potential bias from cancers having dissimilar etiologies and trajectories.

## 5. Conclusions

The results from this study suggest maintained CYP3A activity to the very last days of life, and even a capacity of induction of the enzyme in end-of-life cancer patients. 

Thus, according to the results presented here, dose reduction for drugs metabolized by CYP3A may not be necessary in end-of-life cancer patients. Still, as the results of our study contradict previous findings of CYP3A activity in cancer, they should be interpreted with caution. 

## Figures and Tables

**Table 1:** Demographic data of the 137 cancer patients in the study cohort which were included 1–60 days before death. Median values and interquartile range (IQR) are presented. Statistical analysis was performed with Mann–Whitney test for continuous variables and Fischer’s exact test for binary variables.

**Patient Characteristics**	**Men (*n* = 69)**	**Women (*n* = 68)**	***p*-Value**
Age (years)	70	71	0.90
	(63–78)	(62–78)	
CRP (mg/L)	45	37.5	0.45
	(20–91)	(10–81)	
Albumin (g/L)	24.5	25.5	0.87
	(21–29)	(21–29)	
Creatinine (mmol/L)	72 (59.93)	58 (49–78)	<0.01
Cholesterol (mmol/L)	4.2 (3.6–5.6)	5 (3.6–5.9)	0.22
25-OHD (nmol/L)	41 (27–56)	45 (27–61)	0.64
Survival (days)	31 (16–48)	32 (18–47)	0.64
4β-OHC/C × 10^4^	0.18 (0.13–0.24)	0.19 (0.14–0.32)	0.25
**Type of Cancer**	**Men** (***n*** **= 69**)	**Women** (***n*** **= 68**)	***p*** **-Value**
Lung	12	15	ns
Gastrointestinal	12	7	ns
Pancreas, liver, gallbladder	9	7	ns
Breast	0	14	NA
Urological	5	6	ns
Gynecological	NA	10	NA
Prostate	7	NA	NA
Hematological	5	4	ns
Head-Neck	3	1	ns
Brain tumor	1	1	ns
Esophageal	5	1	ns
Melanoma	5	2	ns
Other (mesothelioma *n* = 4, unknown *n* = 1)	5	0	ns
*n* = 137	69	68	

Abbreviations: 25-hydroxyvitamin D (25-OHD), 4β-hydroxycholesterol/cholesterol (4β-OHC/C), number (*n*), not significant (ns), not applicable (NA), C-reactive protein (CRP).

**Table 2:** Demographic data of the young control populations age 28, 5–35 years (*n* = 280) and elderly control population age 60–85 years (*n* = 30). Median values and interquartile range (IQR) are presented. Statistical analysis was performed with Mann–Whitney test.

Controls: Young	Men (*n* = 107)	Women (*n* = 173)	*p*-Value
Age (years)	27	28	<0.01
	(24–32)	(24–37)	
Cholesterol (mmol/L)	4.1	4.5	<0.01
	(3.7–4.7)	(4.1–5)	
4β-OHC/C × 10^4^	0.14	0.15	0.31
	(0.12–0.18)	(0.12–0.19)	
**Controls: Elderly**	**Men**	**Women**	
	(***n*** **= 15**)	(***n*** **= 15**)	
Age (years)	74 (66–79)	66 (62–74)	<0.01
CRP (mg/L)	1 (1–3)	1 (1–3)	0.57
Albumin (g/L)	37 (34–40)	37 (35–40)	0.57
Creatinine (mmol/L)	89 (81–101)	63 (57–71)	<0.01
Cholesterol (mmol/L)	4,5 (3.8–5)	5.2 (4.5–5.5)	0.12
25-OHD (nmol/L)	70 (66–92)	79 (73–92)	0.21
4β-OHC/C × 10^4^	0.16(0.13–0.17)	0.12(0.10–0.17)	0.53

Abbreviations: 25-hydroxyvitamin D (25-OHD), 4β-hydroxycholesterol/cholesterol ratio (4β-OHC/C), C-reactive protein (CRP).

**Table 3:** The correlation coefficient r (Spearman’s rank test) between 4β-hydroxycholesterol/cholesterol ratio (4β-OHC/C) and the different variables measured in the study. *p* < 0.05 is considered as statistically significant.

Correlation with 4β-OHC/C	All (*n* = 137)	Men (*n* = 69)	Women (*n* = 68)
Age (years)	r = 0.06	r = 0.20	r = −0.08
	*p* = 0.47	*p* = 0.09	*p* = 0.52
Albumin (g/L)	r = −0.02	r = −0.00	r = −0.06
	*p* = 0.78	*p* = 0.98	*p* = 0.64
CRP (mg/L)	r = 0.08	r = −0.03	r = 0.21
	*p* = 0.33	*p* = 0.77	*p* = 0.08
25-OHC (nmol/L)	r = −0.05	r = −0.04	r = −0.10
	*p* = 0.56	*p* = 0.75	*p* = 0.43

Abbreviations: 25-hydroxyvitamin D (25-OHD), 4β-hydroxycholesterol/cholesterol ratio (4β-OHC/C), C-reactive protein (CRP).

## Data Availability

The raw data is available from the corresponding author upon request.
